# BALF Lymphocyte and Cytokine Profiling as Biomarkers of Acute Rejection After Lung Transplantation

**DOI:** 10.3390/jpm15070267

**Published:** 2025-06-23

**Authors:** Silvia Aguado Ibáñez, Carlos Almonacid Sanchez, Piedad Ussetti Gil

**Affiliations:** Lung Transplant Program, Hospital Universitario Puerta de Hierro Majadahonda, 28222 Madrid, Spain; carlos.almonacid@salud.madrid.org (C.A.S.); pied2152@separ.es (P.U.G.)

**Keywords:** acute cellular rejection, transbronchial biopsy, bronchoalveolar lavage, biomarkers, eosinophils, lymphocytes, cytokines

## Abstract

**Background:** Acute cellular rejection (ACR) remains a common complication following lung transplantation and is a major risk factor for chronic lung allograft dysfunction (CLAD). Although transbronchial biopsy (TBB) is the diagnostic gold standard, it is invasive and may be contraindicated in certain patients. This study aimed to assess the diagnostic utility of combining bronchoalveolar lavage fluid (BALF) lymphocyte counts with cytokine profiling—particularly interleukin-17A (IL-17A)—in lung transplant recipients with elevated peripheral blood eosinophil (EOS) counts. **Methods**: We retrospectively analyzed 108 BALF and matched TBB samples from 74 lung transplant recipients with EOS counts >200 cells/μL, collected between 2014 and 2020. BALF lymphocyte percentages and levels of cytokines (IL-4, IL-6, IL-10, IL-13, IL-15, IL-17A, IFN-γ, TNF) were quantified. Associations with histologically confirmed ACR were evaluated using generalized estimating equation models. **Results**: ACR was diagnosed in 57% of TBB samples. BALF lymphocyte percentages were significantly higher in ACR cases (median 8% vs. 4%, *p* < 0.001). Each 1% increase in lymphocytes was associated with a 10% increase in the odds of ACR (OR 1.102; 95% CI 1.076–1.129). IL-17A levels were also significantly elevated in ACR (OR 1.047; 95% CI 1.003–1.092; *p* = 0.032), but with moderate discriminative ability (AUC = 0.629). The combination of BALF lymphocyte counts and IL-17A levels improved diagnostic performance (AUC > 0.76). **Conclusions**: The combined assessment of BALF lymphocyte counts and IL-17A levels in recipients with elevated EOS offers a promising non-invasive strategy to support the diagnosis of ACR. Prospective studies are needed to validate these findings and further refine personalized diagnostic approaches to ACR.

## 1. Introduction

Acute cellular rejection (ACR) is a significant risk factor for the development of chronic allograft dysfunction (CLAD) for lung transplant recipients. The likelihood of developing CLAD is strongly associated with recurrent ACR episodes, greater rejection severity, and late-onset rejection in the post-transplant course [1,2]. Data from the International Society for Heart and Lung Transplantation (ISHLT) showed that up to 30% of lung transplant recipients experienced at least one episode of ACR within the first year post-transplant. Furthermore, ACR accounts for 3.6% of early mortality within the first 30 days and 1.8% within the first year [3].

The high incidence of ACR and its undeniable association with the subsequent development of CLAD highlights the need for strategies that ensure early diagnosis and prompt treatment.

Accurate noninvasive diagnostic markers for ACR are currently lacking, and transbronchial biopsy (TBB) remains the gold standard for diagnosis. Routine surveillance for lung transplant recipients often includes TBB [4]. However, this procedure carries a risk of significant complications, such as hemorrhage (4%) and pneumothorax (2.5%), and may be contraindicated for certain patients [5].

Several studies have sought to identify peripheral blood biomarkers for the early detection of ACR. Among the most studied candidates are various cytokines and chemokines, KL-6, exosomes, and donor-derived cell-free DNA. However, the high cost and technical complexity of many of these methods have limited their integration into routine clinical practice [6,7,8,9].

Complete blood counts are routinely performed as part of the regular follow-up of lung transplant recipients. Some studies have suggested a link between an increase in peripheral blood eosinophil (EOS) counts and acute rejection of the graft. It has been proposed that in immunosuppressed patients, EOS may contribute to ACR through a T-helper 2–mediated immune response [10]. We observed in a previous study that EOS counts could be a useful marker for ACR and could be used to enhance the diagnostic yield of TBB [11].

Bronchoalveolar lavage (BALF) is also routinely performed during the follow-up of lung transplant recipients. The retrieved sample provides valuable insight into the pulmonary microenvironment, allowing for the assessment of cellular composition, cytokine, and protein levels associated with immune response and inflammation [12].

Several authors have investigated the role of different BALF components in the diagnosis of ACR, concluding that the cellular changes in BALF associated with ACR were an increase in neutrophils, lymphocytes, and eosinophils [13,14,15,16,17].

In a previous study, we observed that the simultaneous assessment of BALF lymphocyte counts and EOS counts improved the diagnostic sensitivity of ACR for lung transplant recipients. However, the variability of results previously reported has limited the use of BALF as a diagnostic tool for ACR [18].

Several studies have highlighted the pivotal role of cytokines in the pathogenesis of both CLAD and ACR [19,20]. During ACR episodes, increased levels of pro-inflammatory cytokines, such as interferon-gamma (IFN-γ) and tumor necrosis factor-alpha (TNF-α), have been observed, suggesting their potential utility as diagnostic biomarkers [21]. However, further research is necessary to establish their diagnostic relevance and potential role in improving post-transplant monitoring strategies.

The objective of this study was to assess the diagnostic utility of combining BALF lymphocyte counts and cytokine levels together with elevated EOS for the detection of ACR in lung transplant recipients.

## 2. Materials and Methods

This retrospective study examines the findings from 108 TBB and BALF samples obtained from 74 consecutive lung transplant (LTx) recipients with EOS counts greater than 200 (collected concurrently) between January 2014 and December 2020.

The indications for performing Fiber-optic bronchoscopy (FBC) and obtaining bronchoalveolar samples were categorized as follows:

As part of the scheduled surveillance during the first month post-transplantation.

In response to a significant decline in pulmonary function, defined as a reduction of ≥10% in forced expiratory volume in 1 s (FEV1) compared to the patient’s previous baseline.

For assessment of the resolution of a previously diagnosed acute cellular rejection (ACR) episode following treatment.

In the presence of clinical symptoms or radiological abnormalities suggestive of allograft dysfunction.

During the study period, the immunosuppressive regimen consisted of induction with 20 mg of basiliximab administered on days 0 and 4, followed by triple therapy maintenance. This included tapering doses of methylprednisolone to a maintenance level of 0.10 mg/kg/day, tacrolimus adjusted to maintain blood levels between 5 and 15 ng/mL according to time post-transplantation, and mycophenolate mofetil at doses ranging from 500 to 1000 mg every 12 h depending on individual response and tolerability. Acute rejection episodes were managed with three consecutive intravenous doses of 500 mg of methylprednisolone followed by a tapering course.

The variables collected from the electronic medical records included age, sex, underlying disease, date of transplantation, indication for bronchoscopy, peripheral blood eosinophil count (EOS), corticosteroid dose received 14 days before the procedure, presence and grade of ACR in TBB, presence of eosinophils or infection in BALF, and lymphocyte percentage and cytokine levels in BALF.

The tissue sections from BTB were stained with hematoxylin and eosin, and 2.5-micron-thick sections were prepared at three levels for histological evaluation. The histological diagnosis of acute cellular rejection was classified according to ISHLT recommendations into grade A0 (none), grade A1 (minimal), grade A2 (mild), grade A3 (moderate), and grade A4 (severe) [22].

In the blood samples, we analyzed the absolute EOS count at the time the BALF was performed and the median EOS in the three months before performing the BALF.

Flexible bronchoscopy with transbronchial biopsy (TBB) and BALF sampling was performed in accordance with our national society’s recommendations. After visual inspection of the airways, three 50 mL aliquots of sterile saline were instilled into a segmental bronchus of the middle lobe or lingula. Each aliquot was aspirated using gentle suction to avoid airway collapse. The first 20 mL of the recovered fluid was discarded, and the remaining sample was processed following institutional protocols for microbiological and cytological analysis.

Cellularity in the BAL fluid was determined using 10 mL of a mixture of the three aliquots, filtered through a 70 μm cell strainer and centrifuged at 300× *g* for 10 min. The cell fraction was stained with fluorescence-conjugated monoclonal antibodies labeled with various fluorochromes—fluorescein isothiocyanate (FITC), phycoerythrin (PE), peridinin-chlorophyll protein (PerCP), and allophycocyanin (APC)—targeting the surface antigens CD45, CD14, CD15, HLA-DR, CD19, CD16, CD56, CD3, CD4, CD8, CD25, CD45RO, and CD45RA to identify the different leukocyte subpopulations. Labeled cells were analyzed using a FACSort flow cytometer with CellQuest and Paint-a-Gate Pro software (CellQuest 3.1) (BD Biosciences, San Jose, CA, USA)

Prior to cytokine analysis, infectious processes were systematically ruled out based on clinical evaluation and microbiological cultures of bronchoalveolar lavage (BALF) and bronchial aspirates. Cytokine quantification was performed on thawed BALF samples, measuring soluble inflammatory and anti-inflammatory mediators, including IL-4, IL-6, IL-10, IFN-γ, TNF, IL-17, IL-13, and IL-15. Cytokine concentrations were determined using a quantitative enzyme-linked immunosorbent assay (ELISA) (R&D Systems, Inc., Minneapolis, MN, USA), following the manufacturer’s specifications. In this assay, each well of a pre-coated microplate contained a high-affinity antibody specific to the target cytokine. Serial dilutions of known protein concentrations were incubated alongside patient samples under controlled conditions. Following incubation, wells were washed and further incubated with a peroxidase-conjugated secondary antibody. A subsequent washing step was performed to eliminate unbound reagents before substrate addition, which triggered a colorimetric reaction proportional to the cytokine concentration in each sample. Optical density was measured at 450 nm using a spectrophotometer. A standard calibration curve was constructed by plotting OD values against the known concentrations of reference samples. Cytokine concentrations in patient BALF samples were extrapolated using this standard curve.

Categorical variables were summarized using absolute and relative frequencies, while continuous variables were described using measures of central tendency and dispersion, including median, interquartile range (25th and 75th percentiles), minimum and maximum values, mean, and standard deviation. To explore the relationship between acute cellular rejection (ACR), lymphocyte percentage, and cytokine concentrations in BALF, we considered a variable number of biopsies obtained longitudinally from each patient. Given the repeated measures design and the intra-individual correlation between biopsies, generalized estimating equations (GEEs) were employed. These models accounted for the clustering of observations within patients. A logit link function was applied, with the presence of ACR as the dependent variable and the BALF lymphocyte percentage and cytokine levels as independent predictors.

All statistical analyses were conducted using Stata version 17, with a significance threshold set at *p* < 0.05.

## 3. Results

**Figure 1:**
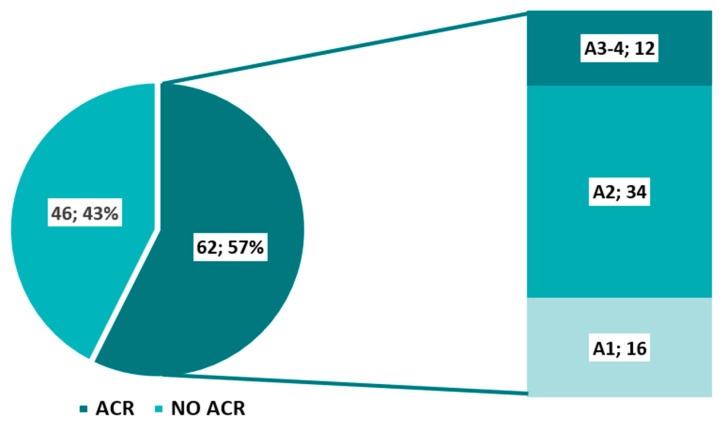
Frequency and grades of ACR. ACR: acute cellular rejection.

A total of 108 TBB and BALF samples were obtained from 74 lung transplant recipients. Evidence of ACR was identified in 57% of the samples (*n* = 62), with grade A2 being the most frequently observed histological finding (Figure 1).

The baseline demographic and clinical characteristics of the study population are presented in Table 1. No significant differences were observed between patients with and without ACR in terms of age, sex, or underlying disease.

**Figure 2:**
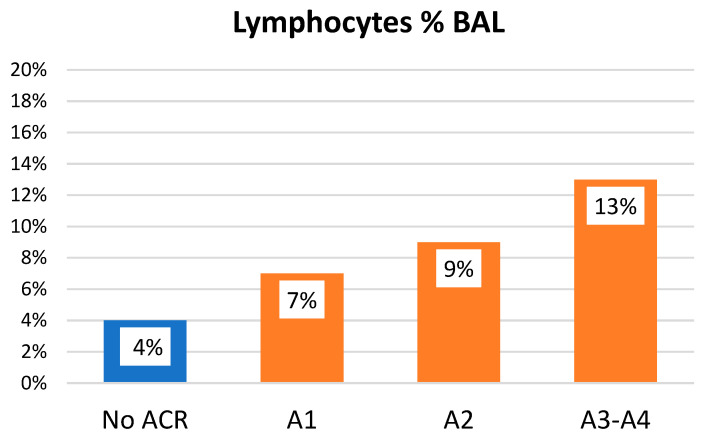
Lymphocytes in BAL According to ACR Grade. BAL: bronchoalveolar lavage; ACR: acute cellular rejection.

No statistically significant differences were observed for neutrophils (ACR: 7 ± 11% vs. No ACR: 13 ± 16%, *p* = 0.106) or macrophages (ACR: 86 ± 14% vs. No ACR: 75 ± 20%, *p* = 0.107). Patients with ACR exhibited significantly higher lymphocyte counts in BALF compared to those without rejection (8% vs. 4%, *p* < 0.001) (Figure 2). Furthermore, the probability of ACR increased by 10% for every 1% increment in BALF lymphocyte count (odds ratio [OR] 1.102; 95% confidence interval [CI] 1.076–1.129), regardless of the underlying disease, indication for bronchoscopy, prior median EOS, presence of eosinophils or infection in BALF, the corticosteroid dose received 14 days before the procedure, and time post-transplant.

Moreover, a positive correlation was observed between the percentage of BALF lymphocytes and the severity of ACR. This association remained significant after adjusting for potential confounders, including underlying disease, bronchoscopy indication, and time post-transplant (OR 1.1387; 95% CI 1.054–1.1395) (Figure 2).

**Figure 3:**
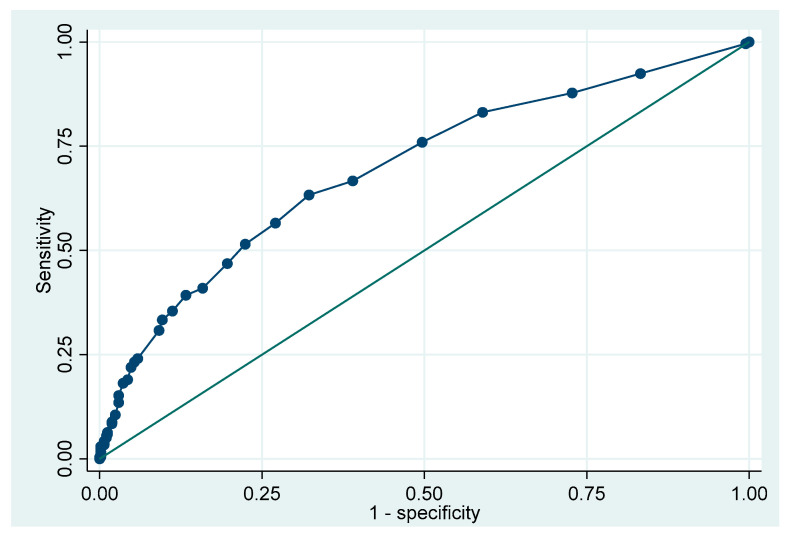
Area under the curve for lymphocytes in the diagnosis of ACR. ACR: acute cellular rejection.

The diagnostic performance of BALF lymphocyte count for ACR was assessed using receiver operating characteristic (ROC) curve analysis, which yielded an area under the curve (AUC) of 0.70. The optimal cutoff point determined by Liu’s method was 6.5%, corresponding to a sensitivity of 63% and specificity of 68% (Figure 3).

Cytokine analysis of BALF samples revealed significantly elevated IL-17A levels in patients with ACR compared to those without rejection (OR 1.047; 95% CI 1.003–1.092; *p* = 0.032). No significant differences were found in the levels of IL-4, IL-6, IL-10, TNF, IFN-γ, IL-13, or IL-15 (Table 2).

**Figure 4:**
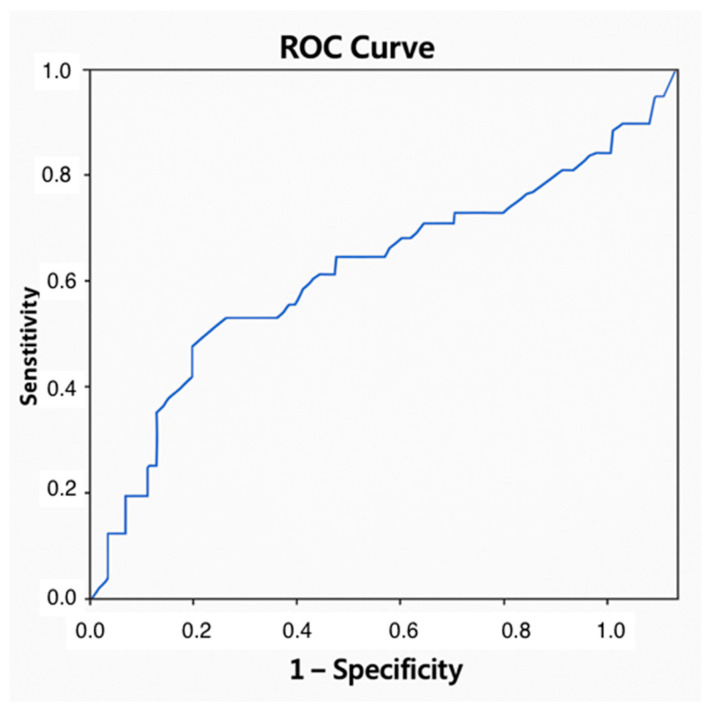
Area under the curve for IL-17A in the diagnosis of ACR. ACR: acute cellular rejection; IL: interleukin.

IL-17A showed a statistically significant association with ACR, but its discriminative capacity was limited (AUC = 0.629) and precluded the establishment of a reliable diagnostic threshold (Figure 4).

**Figure 5:**
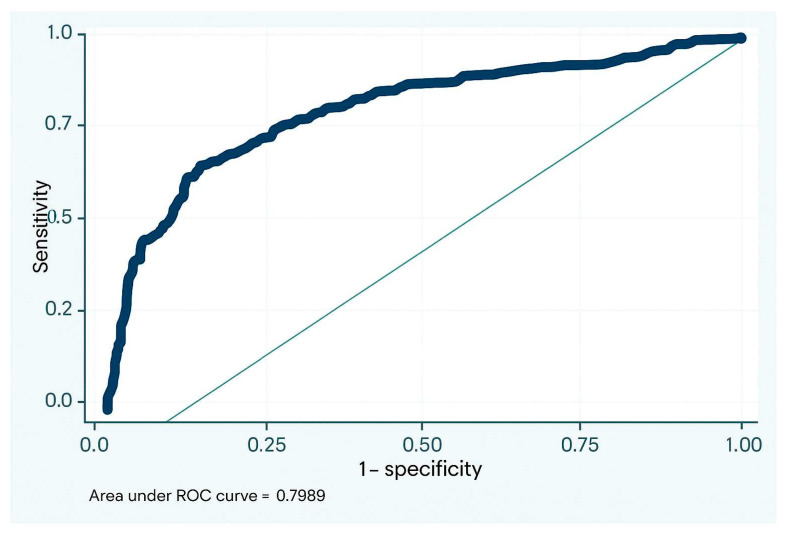
Area under the curve for the diagnosis of ACR combining both biomarkers. ACR: acute cellular rejection.

The combined assessment of lymphocyte count and IL-17A levels in BALF significantly improved the diagnostic performance for ACR with an AUC of 0.76 (Figure 5). This integrative biomarker strategy enhanced diagnostic accuracy regardless of underlying disease, time elapsed since transplantation, the corticosteroid dose received 14 days before the procedure, bronchoscopy indication, and the presence of eosinophils or infection in BALF (OR 1.102 IC95% 1.076-1.129).

## 4. Discussion

We observed that elevated lymphocyte counts and IL-17A levels in BALF are significantly associated with histologically confirmed ACR, particularly in patients with elevated levels of EOS. These findings support the potential utility of BALF profiling as a non-invasive tool for the histopathological evaluation of grafts.

While the role of BALF in post-transplant monitoring has been well established, its diagnostic application in the diagnosis of ACR remains controversial. Our results add to this body of evidence by proposing a dual biomarker strategy that may increase diagnostic reliability [13,14,15,16,17].

The biochemical and cellular analysis of BALF offers important insights into the inflammatory microenvironment of lung allografts. As early as 1975, Achterrath et al. characterized the BALF cellular profile during ACR episodes in animal transplant models [23]. Subsequent studies of human lung transplant recipients have described similar cellular alterations. A meta-analysis of relevant publications identified a BALF neutrophil threshold of ≥12% as a potential marker of ACR, with a reported specificity of 82% and sensitivity of 74%. Additionally, elevated BALF lymphocyte counts have also been associated with ACR, showing acceptable specificity but limited sensitivity [12].

Consistent with prior evidence, a study conducted in our transplant unit identified a BALF lymphocyte count exceeding 12% as a highly specific indicator of ACR [18]. This may support starting treatment for recipients when histological confirmation is unavailable or is inconclusive. Our findings are consistent with previous studies demonstrating a higher percentage of lymphocytes in BALF in the presence of ACR, with a positive correlation between lymphocyte percentage and rejection grade. Moreover, we identified an optimal cutoff value for the diagnosis of ACR, which may aid clinical decision-making. However, its limited sensitivity, also noted by De Hoyos et al., precludes the exclusion of ACR based on this parameter alone. Therefore, patients who fail to respond to empirical therapy should have TBB repeated or alternative diagnostic approaches considered [18,24].

Cytokines are key mediators of ACR, promoting the proliferation, chemotaxis, and activation of cytotoxic T lymphocytes, neutrophils, and alveolar macrophages. Their quantification in BALF may provide relevant diagnostic and prognostic information. IL-6 and IL-8 levels in BALF have been linked to CLAD [25,26], but data on cytokine expression during ACR are more limited and heterogeneous. In our study, among the cytokines analyzed (IL-4, IL-6, IL-10, TNF, IFN-γ, IL-13, IL-15, and IL-17A), only IL-17A showed a statistically significant difference between patients with and without ACR. This suggests that IL-17A may reflect a distinct Th17-driven immunological profile in eosinophilic recipients.

Th17 cells, through the secretion of IL-17A, promote the recruitment and activation of neutrophils, amplifying local inflammation and contributing to allograft tissue injury. The increased levels of IL-17A observed in BALF from patients with ACR may therefore indicate an enhanced Th17-mediated immune response at the site of the allograft. Several studies have suggested that Th17 cells play a role in the pathogenesis of rejection by bridging innate and adaptive immunity and possibly facilitating the transition from acute to chronic rejection. However, the modest discriminative capacity of IL-17A and the lack of a clearly defined diagnostic threshold limit its utility as a standalone biomarker. Our findings are in line with those reported by Vanaudenaerde et al., who associated elevated IL-17A levels with an increased risk of ACR [27]. Similarly, a 2017 meta-analysis identified IL-17 and CXCL10 as the most reliable BALF cytokines associated with ACR. Our negative results for the remaining cytokines are consistent with those reported by Speck et al., who, in a study of more than 700 BALF samples, found no consistent association between IL-6, IL-8, IFN-γ, or TNF-α and ACR [19].Our research suggests that the combination of the percentage of lymphocytes in BALF and IL-17A levels enhances diagnostic performance for ACR. Although further multicenter prospective studies are warranted to validate these results, this integrated biomarker approach may improve clinical suspicion and guide therapeutic decision-making, particularly in complex cases where transbronchial biopsy (TBB) is inconclusive or contraindicated. Nonetheless, the interpretation of these biomarkers should always be contextualized within the clinical framework and complemented by additional diagnostic findings.

The main limitations of our study were its retrospective design and being conducted at a single center, which may have reduced the statistical power of the findings. In relation to the results, while lymphocyte counts in BALF may serve as a useful biomarker for the diagnosis of ACR, the cytokine analysis in BALF did not yield particularly striking results in our cohort. Discrepancies with previous studies may be attributed to differences in BALF collection techniques and cytokine detection methods, notably the volume of saline instilled during the procedure, which can significantly influence the final concentrations of both cellular and soluble mediators.

## 5. Conclusions

The simultaneous assessment of BALF cytokine levels and lymphocyte counts may serve as a valuable tool for ACR diagnosis, particularly in lung recipients with elevated EOS counts. This combined evaluation could be especially beneficial when TBB yields inconclusive results or is not feasible. These findings underline the potential of integrating biomarkers into the diagnostic workflow to facilitate early, non-invasive detection of ACR and enable timely therapeutic interventions. However, further prospective, multicenter studies are necessary to validate these findings and establish standardized thresholds for clinical application.

## Figures and Tables

**Table 1:** Demographic characteristics.

	Total *N* = 74	No ACR *n* = 30; 40.5%	ACR *n* = 44; 59.5%	*p*
Age at transplant (median *, years)	53 (40; 65)	53 (42; 63)	53 (42; 65)	*p* = 0.812
Male (*n*, %)	47 (63.5%)	11 (23.4%)	36 (72.6%)	*p* = 0.291
COPD (*n*, %)	30 (40.5%)	10 (33.3%)	20 (45.5%)	*p* = 0.829
IPF (*n*, %)	16 (21.6%)	7 (23.3%)	9 (20.4%)	
DILD (*n*, %)	16 (21.6%)	5 (16.8%)	11 (25.0%)	
CF (*n*, %)	8 (10.9%)	6 (20.0%)	2 (4.5%)	
Bronchiectasis (*n*, %)	2 (2.7%)	1 (3.3%)	1 (2.3%)	
Other causes (*n*, %)	2 (2.7%)	1 (3.3%)	1 (2.3%)	

(*) Median (p25; p75); ACR: acute cellular rejection; COPD: chronic obstructive pulmonary disease; IPF: idiopathic pulmonary fibrosis; DILD: diffuse interstitial lung disease; CF: cystic fibrosis.

**Table 2:** Cytokines in BAL: overall and by presence or absence of ACR *.

BAL Cytokines	Total	No ACR	ACR	*p*
Human IL-4 (pg/mL)	0 (0; 0)	0 (0; 0)	0 (0; 0)	*p* = 0.959
Human IL-6 (pg/mL)	2.4 (0; 13.16)	0 (0; 7.3)	3.1 (0; 19.0)	*p* = 0.257
Human IL-10 (pg/mL)	0.7 (0.5; 1.2)	0.7 (0.4; 1.1)	0.7 (0.5; 1.2)	*p* = 0.931
Human TNF (pg/mL)	1.0 (0.7; 1.5)	0.9 (0.7; 1.6)	1.1 (0.8; 1.4)	*p* = 0.300
Human IFN (pg/mL)	0.3 (0.2; 0.5)	2.4 (0; 13.16)	0.4 (0.3; 0.5)	*p* = 0.677
IL-17A (pg/mL)	12.7 (8.9; 18.1)	11.5 (8.8; 15.0)	15.5 (8.9; 20.7)	*p* = 0.032 **
IL-13 (pg/mL)	0 (0; 0)	0 (0; 0)	0 (0; 0)	*p* = 0.055
IL-15 (fentogr/mL)	104.8 (80.3; 134.6)	98.3 (80.3; 0132.7	126.6 (70.03; 168.7)	*p* = 0.608

(*) Median (p25; p75); (**) statistically significant; BAL: bronchoalveolar lavage; ACR: acute cellular rejection; IL: interleukin; TNF: tumor necrosis factor; IFN: interferon.

## Data Availability

The datasets generated and analyzed during this study are available from the corresponding author upon reasonable request. Due to privacy and confidentiality considerations, some data may be restricted. Researchers interested in accessing the data should contact s.aguado.nml@gmail.com.

## Abbreviations

The following abbreviations are used in this manuscript:

CLAD	Chronic lung allograft dysfunction
ACR	Acute cellular rejection
ISHLT	International Society for Heart and Lung Transplantation
TBB	Transbronchial biopsy
EOS	Peripheral blood eosinophil
BALF	Bronchoalveolar lavage
IFN-γ	Interferon-gamma
TNF-α	Tumor necrosis factor alpha
LTx	Lung transplant
FBC	Fiber-optic bronchoscopy
FEV1	Forced expiratory volume in 1 s
ELISA	Enzyme-linked immunosorbent assay
